# HDL Proteome in Hemodialysis Patients: A Quantitative Nanoflow Liquid Chromatography-Tandem Mass Spectrometry Approach

**DOI:** 10.1371/journal.pone.0034107

**Published:** 2012-03-21

**Authors:** Alain Mangé, Aurélie Goux, Stéphanie Badiou, Laure Patrier, Bernard Canaud, Thierry Maudelonde, Jean-Paul Cristol, Jérôme Solassol

**Affiliations:** 1 CHU Arnaud de Villeneuve, Dept of Cellular Biology, Montpellier, France; 2 University of Montpellier I, Montpellier, France; 3 Val d'Aurelle Cancer Institute, Dept of Clinical Oncoproteomic, Montpellier, France; 4 CHU Lapeyronie, Dept of Biochemistry, Montpellier, France; 5 UMR 204 NUTRIPASS (University of Montpellier I/II), Montpellier, France; 6 CHU Lapeyronie, Dept of Nephrology, Montpellier, France; National Institutes of Health, United States of America

## Abstract

Aside from a decrease in the high-density lipoprotein (HDL) cholesterol levels, qualitative abnormalities of HDL can contribute to an increase in cardiovascular (CV) risk in end-stage renal disease (ESRD) patients undergoing chronic hemodialysis (HD). Dysfunctional HDL leads to an alteration of reverse cholesterol transport and the antioxidant and anti-inflammatory properties of HDL. In this study, a quantitative proteomics approach, based on iTRAQ labeling and nanoflow liquid chromatography mass spectrometry analysis, was used to generate detailed data on HDL-associated proteins. The HDL composition was compared between seven chronic HD patients and a pool of seven healthy controls. To confirm the proteomics results, specific biochemical assays were then performed in triplicate in the 14 samples as well as 46 sex-matched independent chronic HD patients and healthy volunteers. Of the 122 proteins identified in the HDL fraction, 40 were differentially expressed between the healthy volunteers and the HD patients. These proteins are involved in many HDL functions, including lipid metabolism, the acute inflammatory response, complement activation, the regulation of lipoprotein oxidation, and metal cation homeostasis. Among the identified proteins, apolipoprotein C-II and apolipoprotein C-III were significantly increased in the HDL fraction of HD patients whereas serotransferrin was decreased. In this study, we identified new markers of potential relevance to the pathways linked to HDL dysfunction in HD. Proteomic analysis of the HDL fraction provides an efficient method to identify new and uncharacterized candidate biomarkers of CV risk in HD patients.

## Introduction

In the general population, a decrease in high-density lipoprotein (HDL) cholesterol and/or dysfunctional HDL is recognized as an important risk factor in cardiovascular (CV) diseases [1], [2]. Several studies have focused on the protective effects of HDL on low-density lipoprotein (LDL) oxidation in the arterial wall [3]. HDL is now recognized to protect against plaque formation and progression, in part, through the transport of cholesterol from the peripheral tissues to the liver [4]. The other biological activities of HDL are related to their protein component and are vital in the atheroprotective effects of HDL. Of particular importance are the potent antioxidative and anti-inflammatory activities of HDL, which can directly inhibit LDL oxidation and LDL-induced monocyte infiltration [1], [3]. However, in chronic and acute inflammation, HDL can also have pro-oxidative and proinflammatory properties [1], [5]–[7].

End-stage renal disease (ESRD) patients undergoing chronic hemodialysis (HD) are prone to lipid disorders, chronic inflammation and oxidative stress that contribute to the acceleration of atherosclerosis [8]–[12]. CV diseases are recognized as the major cause of mortality and morbidity in HD patients [15]–[16]. Lipid disorders in HD patients are mainly related to ESRD-induced alterations in lipoprotein metabolism, especially the accumulation of very-low-density lipoproteins (VLDL), a shift in the LDL subclass and alterations in HDL maturation [12], [17]. Patients usually display elevated triglycerides, unchanged or even reduced LDL cholesterol, and decreased HDL cholesterol [13], [14]. In addition, the HD procedure itself can worsen the lipid profile by further decreasing the HDL cholesterol and increasing the LDL cholesterol levels after a session [18]. Aside from a decrease in HDL cholesterol, the CV risk in HD patients is also enhanced by the qualitative abnormalities that lead to dysfunctional HDL [19]–[22].

Because the HDL proteome is involved in HDL functionality, we applied a quantitative proteomics approach to investigate the proteins associated with HDL in HD patients and healthy subjects. We used 8-plex iTRAQ reagents and 2D nanoflow liquid chromatography-mass spectrometry analysis (2D nano-LC/MS/MS) to identify the qualitative and quantitative differences in the proteins purified from the HDL fractions between the HD patients and healthy volunteers. We aimed to identify proteins with a potential relevance in lipid metabolism and the oxidative and inflammatory pathways that could be linked to the HDL dysfunction in HD patients. The identification of new relevant markers could further be used to evaluate the CV risk related to the abnormal HDL proteome.

## Materials and Methods

### Ethics statement

The patients were recruited from the Department of Nephrology using protocols approved by the institutional review board of the University Hospital of Montpellier. Informed consent was obtained from all patients.

### Patient selection

Seven chronic HD patients on renal replacement therapy and seven healthy volunteers were included in the discovery study population. The validation study population consisted of 23 additional sex-matched HD patients and 23 healthy volunteers. The validation study population was sex-matched but not age-matched. The absence of age-matching in the validation study population should be considered a limitation. All HD patients received online hemodiafiltration treatments three times per week, with ultra-pure bicarbonate-based dialysate using a high flux polysulfone membrane (HF-80, Fresenius). Three patients received fibrates, and 13 patients received statins (Table S1). Blood samples were collected on the arterial line before the mid-week dialysis session. All the healthy volunteers were blood donors for the French Blood Agency. Individuals exhibiting acute or chronic infection, hypertension, or diabetes and those requiring medications were excluded. Blood sampling was performed during the blood donation. The plasma was obtained using EDTA-coated tubes, by centrifugation at 3,000 rpm for 15 min, and was stored at −80°C before analysis. All samples were collected during the same month and were subjected to the same number of freeze/thaw cycles (n = 2, before HDL isolation and before proteomic or biochemical analysis).

### Human plasma HDL isolation

The HDL fraction (d = 1.063–1.21 g/ml) from each sample was isolated from the plasma by sequential density ultracentrifugation in three steps using potassium bromide (KBr), as previously described by Morena et al. [22]. Briefly, the VLDL and LDL were sequentially removed by ultracentrifugation at 70,000 rpm for 4 h at 4°C after the adjustment of the plasma density to 1.019 and 1.063 g/ml with KBr, respectively. The HDL fraction was obtained by adjusting the infranatant density to 1.21 g/ml with KBr prior to ultracentrifugation. The HDL fraction was dialyzed against PBS for 24 h at 4°C. Aliquots of the HDL samples were stored at −80°C. The protein concentration was measured in triplicate using the Micro BCA Kit (Pierce). The purity of the HDL fraction was assessed through the measurement of the apolipoprotein (apo) A-I and apoB levels by immunonephelometric assay (Immage 800, Beckman). All samples exhibited an apoB level under the limit of detection (i.e. <0.35 g/L) and an apoA-I level from 1.50 to 5.70 g/L (Table S1).

### Biochemical analyses

The quantification of the total cholesterol, HDL cholesterol, and triglycerides in the plasma and in the HDL fraction was determined using routine assays on the chemistry analyzer Architect C8000 (Abbott). In the HDL fraction, the phospholipid concentration was determined on the same analyzer using reagent from the Diasys Diagnostic System. The quantification of serotransferrin (reagent from Beckman), apoC-II and apoC-III (reagent from Randox), in the HDL fraction, was performed by immunoturbidimetric assay on an AU640 analyzer (Beckman). Haptoglobin, in the HDL fraction, was measured using the AssayMax Haptoglobin ELISA kit (AssayPro). All assays were performed in triplicate. The mean individual data are shown in Table S1.

### iTRAQ reagent labeling and peptide processing

The experimental design used for this study is illustrated in Figure S1. A pool of seven healthy volunteers was used as a reference “control sample”, against which all of the HD patients were compared. By pooling the healthy samples, the potential loss of statistical power arising from a reduced sample size is offset by the reduction in the standard error of the means. iTRAQ labeling was performed according to the manufacturer's protocol (AB Sciex). Briefly, 50 µg of proteins from the HDL control pool and from the seven HD patients was precipitated using four volumes of acetone. The pelleted proteins were dissolved into 500 mM triethylammonium bicarbonate/1% SDS. The proteins were reduced with 5 mM tris-(2-carboxyethyl) phosphine, alkylated with 10 mM methyl methane-thiosulfonate, and trypsin digested overnight. The resulting peptides were labeled for two hours with one of the eight isobaric amine-reactive tags (Figure S1). The labeled peptides were combined and cleaned up using a strong cation exchange cartridge, according to the manufacturer's recommended protocol (AB Sciex). The strong cation exchange eluents were desalted using an Oasis HLB extraction cartridge (Waters Corporation) and vacuum dried before IEF separation.

The peptides were separated using the Agilent 3100 OFFGEL Fractionator (Agilent). Briefly, an IPG Drystrip (24 cm, pH 3–10) was rehydrated for 15 min with 20 µl/well of a solution containing 0.25% IPG buffer, pH 3–10 (GE Healthcare). The desalted peptides were dissolved in the 0.25% IPG buffer, pH 3–10, and the peptide solution (150 µl) was pipetted into each well. Twenty-four well fractionations were focused for 50 kVh, with a maximum current of 50 µA and power of 200 mW. The fractions were purified using OMIX C18 100 µl pipette tips (Varian). The peptides were eluted and lyophilized before being reconstituted for the 2D nano-LC/MS/MS analysis.

### Mass spectrometry analysis of the protein extracts

The peptides were separated using an Ultimate 3000 nano-LC system coupled to a Probot™Microfraction Collector (Dionex). The nano-LC separations were performed using an Acclaim PepMap™ (C18, 3 µm, 100 Å) 75 µm/15 cm column. The mobile phases used were 2% acetonitrile (ACN) with 0.05% trifluoroacetic acid (TFA) (A) and 80% ACN with 0.05% TFA (B). The gradient elution steps were performed with a flow rate of 0.3 µl/min as follows: 0–50% B over 60 min, 50–80% B over 30 min, 80–100% B over 5 min, and then 100% B for an additional 10 min. The fractions were mixed directly with the matrix-assisted laser desorption/ionization (MALDI) matrix solution (2 mg/ml α-cyano-4-hydroxycinnamic acid in 70% ACN with 0.1% TFA) at a flow rate of 1.2 µl/min. Forty fmol of human [Glu1] Fibrinopeptide B (*m/z* 1570.57) was spiked into each spot as an internal standard. Spotting onto the Opti-TOF™ LC/MALDI insert plates (AB Sciex) was performed using the Probot spotting device over a 110-min period at a speed of 11 s per well.

The plates were analyzed using a MALDI time of flight (MALDI TOF/TOF) 4800 mass spectrometer (AB Sciex). MS spectra from *m/z* 700 to 4000 were acquired in positive reflector ion mode using 750 laser shots. The ten most abundant peptide precursor ions, with a signal-to-noise ratio greater than or equal to 50, were selected for MS/MS analysis using 2500 laser shots from *m/z* 300 to 1500.

### Data analysis and interpretation

The protein identification and quantification were performed with the ProteinPilot™ Software 2.0.1 (AB Sciex). The MS/MS spectra obtained were searched against the UniProt database (UniProtKB release 2009_09). The search parameters for tryptic cleavage and accuracy are built-in functions of the software. The other data analysis parameters were as follows: 8-plex iTRAQ peptide labeling, cys-alkylation by MMTS, biological modifications, a thorough identification search, and a minimum detected protein threshold of 1.3 (which corresponds to a confidence score of 95%). The unused protein score, calculated by ProteinPilot, is a measurement of the confidence in the protein identification after taking into account all of the peptide data for a given protein and excluding any evidence that is better explained by a higher ranking protein. Proteins comprising one or more peptides with a high confidence score (>95%) and a low false discovery rate (FDR) (estimated local FDR of 5%) were considered positively identified. A reversed-decoy database from the same UniProt database was used to estimate the local FDR using the Proteomics System Performance Evaluation Pipeline (PSPEP) tool [23]. The use of a 5% local FDR indicates that each protein identified has at least a 95% probability of being correct.

The relative quantification was based on the ratio of the areas under the reporter peaks, from the control samples (*m/z* 113 Da) and the patient samples (*m/z* 114, 115, 116, 117, 118, 119, and 121 Da). The following criteria were required to select a protein for further analysis: two or more unique peptides with a high confidence score (>95%), a *p*-value for protein quantification <0.05, greater than a 1.3-fold difference relative to the control sample, and detection of the protein in more than 50% of the HD patient samples (at least 4 of the 7 HD patient samples). For more information about the quantification methodology, see the Text S1.

### Statistical analysis

The results are expressed as the mean ± standard error of the mean (SEM). The statistical analyses were performed using the Mann-Whitney *U* test using GraphPad InStat. A *p*-value of <0.05 was chosen for statistical significance. The enrichment analysis of the gene ontology (GO) was determined using the DAVID bioinformatics resources [24]. The significance of the gene-term enrichment was determined using a modified Fischer's exact test (EASE score), which ranks the overrepresented GO processes.

## Results

### Proteomic HDL analysis of the discovery population

The characteristics of the patients, for both study populations, are summarized in Table 1 and Table S1. In the discovery and in the validation set, the HD patients exhibited significantly lower cholesterol levels than the healthy subjects, including lower HDL cholesterol and lower LDL cholesterol levels. There was no difference in the plasma triglyceride levels between the HD and healthy subjects, in either the discovery or the validation set.

**Table 1:** Clinical characteristics of the study population.

	Discovery set			Validation set		
	Healthy n = 7	HD n = 7	*p*-value*	Healthy n = 23	HD n = 23	*p*-value*
**Gender**	3M/4F	3M/4F	NS	15M/8F	15M/8F	NS
**Age (years)**	59.7±0.36	69.1±3.59	NS	56.6±0.76	69.7±3.01	<0.05
**Plasma (mmol/L, mean ± SEM)**						
**Total cholesterol**	5.4±0.44	3.7±0.38	<0.05	5.0±0.23	3.7±0.21	<0.05
**Triglyceride**	1.5±0.25	1.5±0.37	NS	1.3±0.11	1.5±0.19	NS
**HDL-Cholesterol**	1.7±0.13	1.1±0.23	<0.05	1.4±0.07	1.0±0.07	<0.05
**LDL-Cholesterol**	3.0±0.35	1.8±0.24	<0.05	2.9±0.20	1.9±0.13	<0.05
**HDL (mmol/L, mean** ± **SEM)**						
**Total cholesterol**	4.8±0.41	5.4±1.01	NS	4.7±0.43	5.2±0.40	NS
**Triglyceride**	0.4±0.05	0.5±0.09	NS	0.4±0.05	0.7±0.13	<0.05
**Phospholipids**	3.7±0.22	4.5±0.56	NS	3.7±0.29	4.5±0.32	<0.05
**Total lipids**	8.9±0.60	10.4±1.58	NS	8.8±0.75	10.5±0.75	NS

* Mann-Whitney test.

Using iTRAQ labeling and 2D nano-LC/MS/MS, a total of 303 non-redundant proteins were identified and quantified for the comparison between the seven individual HD patients and the seven healthy volunteers. Among these proteins, 122 were further selected using stringent criteria, such as containing one or more peptides with a >95% confidence score and a 5% local FDR. The complete MS and MS/MS data are shown in Table S2 and Table S3.

The iTRAQ peptide labeling efficiency was 98.8%. Only 40 of the 122 proteins showed a differential expression in the HD patients that also corresponded to our selection criteria (identified and quantified in at least four of the seven HD patients, a differential expression of at least 1.3-fold relative to the control samples, and a *p*-value<0.05). The complete list of the proteins defined by these criteria and confidently identified, containing at least two peptides with a high confidence score, is shown in Table 2. Among the 40 selected proteins, more than 68% were found to be differentially expressed in at least six of the HD patients (10 were upregulated and 17 were downregulated) compared with the healthy subjects.

**Table 2:** iTRAQ ratios for the healthy and HD samples: HD patient samples (HD1-7) *vs.* pooled healthy subject samples.

Uniprot accession n°	Gene symbol	Protein name	HD 1	HD 2	HD 3	HD 4	HD 5	HD 6	HD 7
**Upregulated**									
P02652	APOA2	Apolipoprotein A-II	**1.86**	**3.31**	**2.86**	**1.34**	**2.57**	**2.45**	**2.30**
P02656	APOC3	Apolipoprotein C-III	**3.24**	**3.11**	**2.65**	**2.84**	**3.65**	**3.70**	**1.94**
P02760	AMBP	Protein AMBP	**1.41**	**1.97**	**1.58**	**1.85**	**1.94**	**1.91**	**1.42**
P05090	APOD	Apolipoprotein D	**1.51**	**1.41**	**1.30**	**1.56**	**1.72**	**1.63**	**1.49**
P02655	APOC2	Apolipoprotein C-II	**2.64**	**2.97**	**2.87**	**3.00**	**4.42**	**4.43**	**2.11**
P35542	SAA4	Serum amyloid A-4 protein	**1.99**	**2.35**	**1.54**	**1.53**	**1.52**	**2.76**	**1.80**
P08519	LPA	Apolipoprotein(a)	**2.81**	**1.60**	**1.43**	**2.83**	*1.14*	**2.81**	**1.79**
P02753	RBP4	Retinol-binding protein 4	**1.80**	**2.18**	*1.25*	**1.96**	**1.45**	**1.60**	**1.59**
P02654	APOC1	Apolipoprotein C-I	*1.37*	**1.79**	**2.05**	**1.54**	**1.96**	**2.49**	**1.84**
P04180	LCAT	Phosphatidylcholine-sterol acyltransferase	**2.46**	**1.72**	**1.57**	**1.39**	**1.54**	**1.74**	*1.33*
P06727	APOA4	Apolipoprotein A-IV	*1.12*	**2.05**	**1.39**	**2.55**	*1.20*	**1.71**	**1.54**
P02649	APOE	Apolipoprotein E	*1.01*	**1.49**	*1.09*	2.94	1.34	2.62	1.81
P02735	SAA1	Serum amyloid A protein	**1.42**	**1.32**	**1.32**	*0.80*	**8.90**	**3.43**	*0.96*
O95445	APOM	Apolipoprotein M	**1.66**	**1.44**	*1.28*	**1.58**	**1.31**	**1.40**	*1.14*
P27169	PON1	Serum paraoxonase/arylesterase 1	**2.32**	**1.47**	*0.97*	**1.56**	**1.98**	*1.10*	**1.60**
P55056	APOC4	Apolipoprotein C-IV	**1.35**	**2.16**	**1.30**	*1.99*	**2.27**	**2.88**	*0.83*
O14791	APOL1	Apolipoprotein L1	**1.78**	**2.00**	*1.19*	*0.91*	**1.31**	**1.33**	*1.02*
P04114	APOB	Apolipoprotein B-100	**1.41**	**1.39**	*1.10*	*1.21*	**1.98**	**1.56**	*1.10*
P61769	B2M	Beta-2-microglobulin	*2.14*	**3.68**	**3.73**	*2.12*	3.69	3.74	*3.29*
**Downregulated**									
P02787	TF	Serotransferrin	**0.36**	**0.36**	**0.43**	**0.57**	**0.32**	**0.43**	**0.48**
P01024	C3	Complement C3	**0.65**	**0.54**	**0.65**	**0.71**	**0.55**	**0.64**	**0.58**
P02675	FGB	Fibrinogen beta chain	**0.47**	**0.50**	**0.39**	**0.51**	**0.33**	**0.36**	**0.38**
P00738	HP	Haptoglobin	**0.39**	**0.27**	**0.33**	**0.44**	**0.37**	**0.32**	**0.47**
P01876	IGHA1	Ig alpha-1 chain C region	**0.34**	**0.33**	**0.32**	**0.62**	**0.36**	**0.36**	**0.41**
P01023	A2M	Alpha-2-macroglobulin	**0.24**	**0.19**	**0.25**	**0.25**	**0.20**	**0.24**	**0.22**
P02679	FGG	Fibrinogen gamma chain	**0.58**	**0.37**	**0.33**	**0.54**	**0.35**	**0.39**	**0.39**
P08603	CFH	Complement factor H	**0.47**	**0.32**	**0.48**	**0.64**	**0.37**	**0.42**	**0.48**
P01842	IGLC1	Ig lambda chain C regions	**0.45**	**0.37**	**0.59**	**0.60**	**0.35**	**0.36**	**0.53**
P01871	IGHM	Ig mu chain C region	**0.45**	**0.22**	**0.30**	**0.50**	**0.28**	**0.26**	**0.33**
P02751	FN1	Fibronectin	**0.44**	**0.34**	**0.34**	**0.57**	**0.32**	**0.29**	**0.36**
P02671	FGA	Fibrinogen alpha chain	**0.49**	*1.02*	**0.55**	**0.69**	**0.40**	**0.44**	**0.50**
P02790	HPX	Hemopexin	**0.46**	**0.46**	**0.53**	*0.81*	**0.47**	**0.68**	**0.64**
P01042	KNG1	Kininogen-1	**0.57**	**0.71**	**0.75**	*0.92*	**0.43**	**0.77**	**0.66**
P00734	F2	Prothrombin	**0.47**	**0.46**	**0.56**	**0.70**	**0.52**	*0.77*	**0.73**
P04196	HRG	Histidine-rich glycoprotein	**0.38**	**0.53**	**0.49**	*0.88*	**0.37**	**0.53**	**0.58**
Q14624	ITIH4	Inter-alpha-trypsin inhibitor heavy chain H4	*0.69*	**0.39**	**0.55**	0.60	**0.35**	**0.58**	**0.58**
P04004	VTN	Vitronectin	**0.60**	**0.61**	**0.54**	*0.71*	**0.48**	*0.66*	**0.65**
P01008	SERPINC1	Antithrombin-III	**0.64**	**0.68**	*0.85*	*1.00*	**0.67**	**0.73**	*0.82*
P10909	CLU	Clusterin	**0.67**	**0.58**	**0.77**	*1.09*	**0.62**	*0.93*	*0.86*
P01700	LV102	Ig lambda chain V-I region HA	*0.42*	**0.45**	**0.40**	*0.49*	**0.21**	*0.25*	**0.38**

Ratios of at least 1.3-fold relative to the control samples, and a *p*-value<0.05 are indicated in bold.

The biological processes and the molecular functions of all of the proteins identified in the HDL fraction were classified using a GO classification system and are presented in Table S4. The proteins were associated with a broad range of molecular functions such as lipid and cholesterol transport, lipid metabolism, inflammatory response, the complement and coagulation cascade, the response to metal ions, hemostasis, and endopeptidase inhibitory activity. We investigated the enrichment of the 40 differentially expressed proteins using the functional classification tool. Table 3 shows the GO categories associated with the 40 differentially expressed proteins. Individual mapping of the upregulated and downregulated proteins shows that the lipid/cholesterol transport and metabolism proteins were the most significant functional processes represented by the upregulated proteins, whereas the acute inflammatory response, complement activation, and endopeptidase inhibitor activity were the most significant functional processes represented by the downregulated proteins.

**Table 3:** The gene ontology category enrichments for the differentially expressed proteins in the HDL fraction.

Gene Ontology Category	*P*-value*	iTRAQ expression	Gene symbol
Acute inflammatory response (GO:0002526)	4.00E-11	Down-regulated	FN1. TF. KNG1. ITIH4. CFH. CLU. C3. A2M. F2
Endopeptidase inhibitor activity (GO:0004866)	9.00E-07	Down-regulated	HRG. KNG1. ITIH4. SERPINC1. C3. A2M
Heparin binding (GO:0008201)	2.10E-06	Down-regulated	HRG.FN1. SERPINC1. VTN
Complement and coagulation cascade (KEGG hsa04610)	3.30E-14	Down-regulated	FGG. FGA. KNG1. FGB. SERPINC1. CFH. C3. A2M. F2
Cation homeostasis (GO:0055080)	3.80E-04	Down-regulated	TF. KNG1. HP. HPX. F2
Lipid transport (GO:0006869)	5.00E-26	Up-regulated	RBP4. APOB. APOA4. LCAT. LPA. APOL1. APOE.APOC2. APOC3. APOM. APOA2. APOC4. APOC1
Cholesterol transport (GO:0030301)	2.70E-19	Up-regulated	APOB. APOA4. LCAT. APOE. APOC2. APOC3. APOM.APOA2. APOC1
Cholesterol metabolic process (GO:0008203)	2.40E-13	Up-regulated	APOB. APOA4. LCAT. APOL1. APOE. APOC3. APOA2.APOC1
Lipid metabolic process (GO:0019216)	1.20E-12	Up-regulated	APOB. APOA4. APOE. APOC2. APOC3. APOM. APOA2.APOC1
Regulation of lipoprotein oxidation (GO:0034442)	5.60E-06	Up-regulated	PON1. APOA4. APOM

* Enrichment *P*-values were calculated using a modified Fischer's exact test (EASE score).

### Confirmation of the differential levels of HDL-associated proteinsin the discovery study population by biochemical analysis

**Figure 1:**
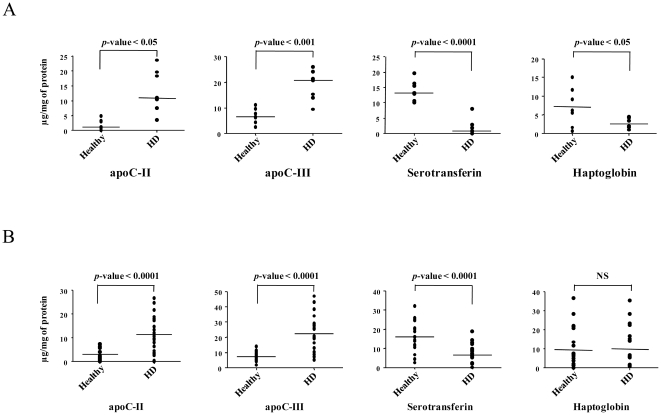
The expression analysis of apoC-II, apoC-III, serotransferrin and haptoglobin in the HDL fractions of the healthy volunteers and the HD patients. The concentration of the proteins in the 14 samples of the discovery set (A) and in the 46 samples of the validation set (B) were determined using biochemical analyses. The mean is indicated by the horizontal line.

A panel of four proteins identified by MS analysis in all seven HD patients (two upregulated [apoC-II and apoC-III] and two downregulated [serotransferrin and haptoglobin]) was selected to confirm the changes in the protein expression that was observed in the proteomic analysis. These four proteins were quantified in the same population using biochemical or immunological assays. The results were in agreement with the MS findings, and we showed a significant correlation between the iTRAQ and the biochemical quantification ratio for the four proteins (Table 4). There were no significant differences between the iTRAQ and the biochemical quantification ratios, except for apoC-II and serotransferrin (Table 4). The comparison of the iTRAQ reporter ion signal intensities revealed a 3.11-fold increase and a 0.41-fold decrease in apoC-II and serotransferrin, respectively, in the HD patients as compared with the control pool. These results are consistent with the 6.53-fold increase and 0.14-fold decrease, respectively, detected by the biochemical quantification using the same samples, even if the magnitude of observed change is far greater than that seen in the iTRAQ assay (*p*-value<0.05 and *p*-value<0.005 for apoC-II and serotransferrin, respectively). When we compared the expression of each protein, the expression of apoC-II and apoC-III were significantly higher in the HDL fraction of the HD patients when compared with the healthy volunteers whereas serotransferrin and haptoglobin showed significantly lower expression in the HDL fraction of the HD patients when compared with the healthy volunteers (Figure 1A).

**Table 4:** Comparison of the iTRAQ ion ratios and the biochemical quantification ratios.

Protein name	Ratio	HD 1	HD 2	HD 3	HD 4	HD 5	HD 6	HD 7	Mean	Correlation	
										Pearson r	*p*-value
Apolipoprotein C-II	iTRAQ	2.64	2.97	2.87	3	4.42	4.43	2.11	3.11	0.89	0.008
	Quant	5.12	5.3	3.66	8.89	9.53	11.51	1.71	6.53		
Apolipoprotein C-III	iTRAQ	3.24	3.11	2.65	2.84	3.65	3.7	1.94	2.96	0.85	0.03
	Quant	1.38	3.54	2.23	3.02	3.11	3.8	2.03	2.73		
Serotransferrin	iTRAQ	0.36	0.36	0.43	0.57	0.32	0.43	0.48	0.41	0.90	0.006
	Quant	0	0	0.05	0.56	0	0.2	0.13	0.14		
Haptoglobin	iTRAQ	0.39	0.27	0.33	0.43	0.37	0.32	0.47	0.36	0.89	0.02
	Quant	0.22	0.14	0.29	0.48	0.62	0.27	0.59	0.33		

### Differential levels of the HDL-associated proteins in an independent validation study population

We quantified apoC-II, apoC-III, serotransferrin and haptoglobin in the HDL fraction of 23 additional healthy control volunteers and 23 sex-matched HD patients (Table 5 and Figure 1B). The apoC-II in the HDL fraction was confirmed to be significantly higher in the HD patients (11.4 µg/mg of protein, 95% CI [8.1–14.8]), when compared with the healthy controls (3.1 µg/mg of protein, 95% CI [2.0–4.2]). A similar result was obtained with apoC-III, which was found to be significantly higher in the HD patients (22.5 µg/mg of protein, 95% CI [17.4–27.6]) when compared with the healthy controls (7.5 µg/mg of protein, 95% CI [6.1–8.9]). Therefore, the apoC-II/apoC-III ratio was also found to be significantly higher in the HD patients (ratio of 0.42) when compared with the healthy controls (ratio of 0.25). The serotransferrin levels in the HDL fraction were significantly lower in the HD patients (6.7 µg/mg of protein, 95% CI [4.5–8.9]) when compared with the healthy controls (16.4 µg/mg of protein, 95% CI [13.1–19.7]). In contrast, there were no confirmed differences in the haptoglobin levels between the healthy controls (9.5 µg/mg of protein, 95% CI [5.2–13.8]) and the HD patients (9.8 µg/mg of protein, 95% CI [5.4–14.2]). Neither age nor the lipid-lowering drugs taken by some patients were shown to have a significant impact on our validation data (Table 5).

**Table 5:** The differential expression levels of apoC-II, apoC-III, serotransferrin and haptoglobin in the independent healthy and HD validation study population.

	Healthy		HD		*p*-value*
	N	Concentration	N	Concentration	
		(µg/mg of protein)		(µg/mg of protein)	
**Apolipoprotein C-II**	23	3.1, 95% CI [2.0–4.2]	23	11.4, 95% CI [8.1–14.8]	<0.0001
**Age≤56** a	8	3.0, 95% CI [1.2–4.8]	6	8.2, 95% CI [3.1–13.3]	<0.05
**Age>56** a	15	3.2, 95% CI [1.6–4.7]	17	12.6, 95% CI [8.2–17.0]	<0.05
**No Treatment** b	-	3.1, 95% CI [2.0–4.2]	11	9.8, 95% CI [4.6–15.1]	<0.05
**With Treatment** b	-	3.1, 95% CI [2.0–4.2]	12	13.2, 95% CI [8.3–18.1]	<0.0001
**Apolipoprotein C-III**	23	7.5, 95% CI [6.1–8.9]	23	22.5, 95% CI [17.4–27.6]	<0.0001
**Age≤56** a	8	6.3, 95% CI [3.4–9.3]	6	20.5, 95% CI [11.8–29.1]	<0.05
**Age>56** a	15	8.1, 95% CI [6.5–9.8]	17	23.2, 95% CI [16.6–29.9]	<0.05
**No Treatment** b	-	7.5, 95% CI [6.1–8.9]	11	20.7, 95% CI [13.3–28.0]	<0.001
**With Treatment** b	-	7.5, 95% CI [6.1–8.9]	12	24.5, 95% CI [16.3–32.7]	<0.0001
**Serotransferrin**	23	16.4, 95% CI [13.1–19.7]	23	6.7, 95% CI [4.5–8.9]	<0.0001
**Age≤56** a	8	17.8, 95% CI [12.8–22.7]	6	8.0, 95% CI [3.1–13.0]	<0.05
**Age>56** a	15	15.6, 95% CI [10.9–20.3]	17	6.2, 95% CI [3.5–8.9]	<0.001
**No Treatment** b	-	16.4, 95% CI [13.1–19.7]	11	6.9, 95% CI [4.1–9.9]	<0.001
**With Treatment** b	-	16.4, 95% CI [13.1–19.7]	12	6.4, 95% CI [2.4–10.3]	<0.001
**Haptoglobin**	23	9.5, 95% CI [5.2–13.8]	23	9.8, 95% CI [5.4–14.2]	NS
**Age≤56** a	8	10.3, 95% CI [1.9–18.6]	6	9.0, 95% CI [1.5–16.4]	NS
**Age>56** a	15	9.0, 95% CI [3.2–14.7]	17	10.0, 95% CI [4.2–16.0]	NS
**No Treatment** b	-	9.5, 95% CI [5.2–13.8]	11	6.6, 95% CI [2.2–10.9]	NS
**With Treatment** b	-	9.5, 95% CI [5.2–13.8]	12	13.0, 95% CI [5.0–21.1]	NS

* Mann-Whitney test.

a Healthy and HD patient groups were divided into patients under the age of 56 and patients above the age of 56.

b HD patient group was divided into patients treated with lipid-lowering drugs and patients without treatment, and each HD subgroup was compared with the healthy patient group.

## Discussion

In this study, we investigated the HDL proteome in HD patients who are considered to have a high risk for CV diseases. Multidimensional nano-LC/MS/MS was used for the analysis of the HDL samples, which were labeled with isobaric mass tags (iTRAQ) to identify the proteins that were differentially expressed between the HDL fractions of the HD patients and the healthy volunteers. Earlier studies have identified approximately 50 distinct proteins associated with the HDL fraction, using 2D electrophoresis and mass spectrometry approaches [19], [25]–[29]. Notably, in our 2D nano-LC/MS/MS approach, we greatly improved the HDL fraction proteome coverage by identifying 122 proteins associated with the HDL fraction, including 80–90% of the previously identified proteins [19], [25]–[30]. The proteins associated with HDL have been implicated in a wide range of functions, such as lipid metabolism, inflammatory response, the complement and coagulation cascade, and the activity of the endopeptidase inhibitor. Among the proteins involved in lipoprotein metabolism, we detected several apolipoproteins and enzymes that are already known to be associated with HDL, such as apoA-I, apoA-II, apoA-IV, apoC-I, apoC-II, apoC-III, apoC-IV, apoD, apoE, apoJ, apoL-I, apoM, lecithin cholesterol acyl transferase, and the cholesterol ester transfer protein. In addition, we identified other proteins associated with the HDL fraction that are related to the inflammation and oxidative pathways, such as the serum amyloid proteins, paraoxonase-1 and 3, α1-antitrypsin, and several complement component proteins.

We identified 40 differentially expressed proteins, with a high confidence score, between the HD patients and the healthy volunteer samples; of these proteins, four proteins that were differentially expressed in all seven HD patients and that were previously shown to be associated with HDL [28], [31] were used to validate the proteomic analysis. The validation step confirmed the increase of apoC-II and apoC-III in the HDL fraction of HD patients compared with the healthy controls. Both apoC-II and apoC-III were elevated in the HDL fraction of the HD patients, leading to an increase in the apoC-II/apoC-III ratio. This observation is surprising when considering the respective functions of apoC-II and apoC-III, which are to activate and inhibit lipoprotein lipase activity, respectively [32]. VLDL accumulation related to impaired lipoprotein lipase activity and a decrease in the plasma apoC-II/apoC-III ratio are classically observed in HD patients [12]. Nevertheless, the increase of both apoC-II and apoC-III in the HDL fraction of the HD patients does not predict the activity of lipoprotein lipase for VLDL delipidation, because the active form of apoC-II or apoC-III are carried by VLDL [33]. It can be hypothesized that the increase of apoC-II and apoC-III in the HDL fraction is due to an abnormal transfer of apoC to the VLDL and the chylomicrons. This accumulation of apoC-II and apoC-III in the HDL fraction could be a marker of impaired maturation of the HDL particles, leading not only to dysfunctional HDL and a potential impairment of reverse cholesterol transport but also to an overall alteration of lipoprotein metabolism through modification in the exchange of apoC. Recent studies have demonstrated that an increase in the plasma apoC-III levels was positively associated with the smaller sizes of the HDL particles, and this association was even higher when the elevation of apoC-III and apoC-II occurred simultaneously [34]. Another study, performed on HD patients, demonstrated a relationship between a change in the HDL proteome, including an increase of apoC-III, and a change in the *in vitro* HDL functionality, as assessed by their ability to promote cholesterol efflux in lipid-laden macrophages [19]. Oxidative stress and inflammation, which are frequently observed in HD patients, could contribute to the formation of dysfunctional HDL, as suggested by *in vitro* studies [35], [36]. Finally, concordant to our study, dysfunctional HDL and the impairment of reverse cholesterol transport have been shown to be key features of dyslipidemia in HD and have been shown to contribute to the high risk for CV diseases [12], [19]–[22].

The atheroprotective effects of HDL are mediated, aside from the reverse cholesterol transport function, by the antioxidative and anti-inflammatory properties of HDL [2], [4], [5]. For example, HDL may prevent LDL oxidation, a key event in the initiation and progression of vascular atherosclerotic lesions [3]. Interestingly, impairment of the HDL antioxidant properties in HD patients was previously demonstrated using an *in vitro* LDL oxidation model [21], [22]. Among the processes involved in LDL oxidation, metal cations, such as iron or copper, may promote the initial step of lipid peroxidation [37]. Our study identified a cluster of proteins involved in metal cation homeostasis, such as haptoglobin and serotransferrin, for which the expression was decreased in the HDL fraction of the HD patients compared with the healthy volunteers. In agreement with the previous data shown in plasma [38], the haptoglobin levels in the HDL fraction of the HD patients appear downregulated when compared with the controls in our discovery study population. Haptoglobin, an acute phase protein, avidly binds to hemoglobin that is released into the plasma during physiological and pathological hemolysis and prevents the iron- and heme-mediated oxidative side effects [39], [40]. Unfortunately, the validation step in our independent study population did not confirm the significant difference between the groups. However, the haptoglobin levels should be interpreted with caution in the HD patients, since many conditions such as hemolysis, folate deficiency, liver disease, anemia, infection or inflammation can lead to haptoglobin variations. These confounding factors could explain the dissociation observed for the haptoglobin levels between the discovery and the validation study populations. Serotransferrin, the major iron binding protein, transports iron between the sites of absorption, storage, and utilization and delivers the iron required for cellular processes [41]. Interestingly, serotransferrin has already been shown to be associated with HDL, using both western blot and proteomic analyses [28], [42]. Notably, the *in vitro* oxidation of LDL by copper was inhibited by HDL containing serotransferrin and ceruloplasmin. In contrast, the removal of the lipoproteins containing serotransferrin or ceruloplasmin reduced the ability of the lipoproteins to inhibit the oxidation of LDL [42]. Therefore, it could be hypothesized that the decrease of serotransferrin in the HDL fraction of the HD patients leads to decreased protection against LDL oxidation.

In conclusion, we showed that HD patients exhibit major modifications of their HDL proteome, in addition to the quantitative decrease of HDL cholesterol. When comparing a fixed amount of HDL-associated proteins, 40 proteins, primarily involved in lipid metabolism, the inflammation pathway, complement activation, and metal cation homeostasis, appear to be differentially expressed between the HD patients and the healthy volunteers. The expression level of apoC-II, apoC-III, and serotransferrin was validated in an independent population set, improving the reliability of our findings. Note that chronic HD is a complex therapy, and the comparison of the HDL proteome between patients undergoing HD and healthy subjects does not allow us to distinguish the effects of ESRD and HD itself. Although further investigations with a larger number of subjects will be needed to determine whether the proteins identified in this study can be used as relevant CV-risk biomarkers, we identified proteins with a potential relevance to the pathways linked to HDL dysfunction in chronic HD patients.

## Supporting Information

Figure S1 Experimental design.
(TIF)

Table S1 Clinical characteristics of the study population.
(XLS)

Table S2 Protein summary report generated by ProteinPilot.
(XLS)

Table S3 Peptide summary report generated by ProteinPilot.
(XLS)

Table S4 Enriched biological process and molecular function using the gene ontology classification (http://david.abcc.ncifcrf.gov).
(XLS)

Text S1 (DOC)
