# Supplementary figures and images for: HDL Proteome in Hemodialysis Patients: A Quantitative Nanoflow Liquid Chromatography-Tandem Mass Spectrometry Approach
Source: PLoS One. 2012 Mar 21;7(3):e34107. doi: 10.1371/journal.pone.0034107 (PMC3309955; doi:10.1371/journal.pone.0034107)

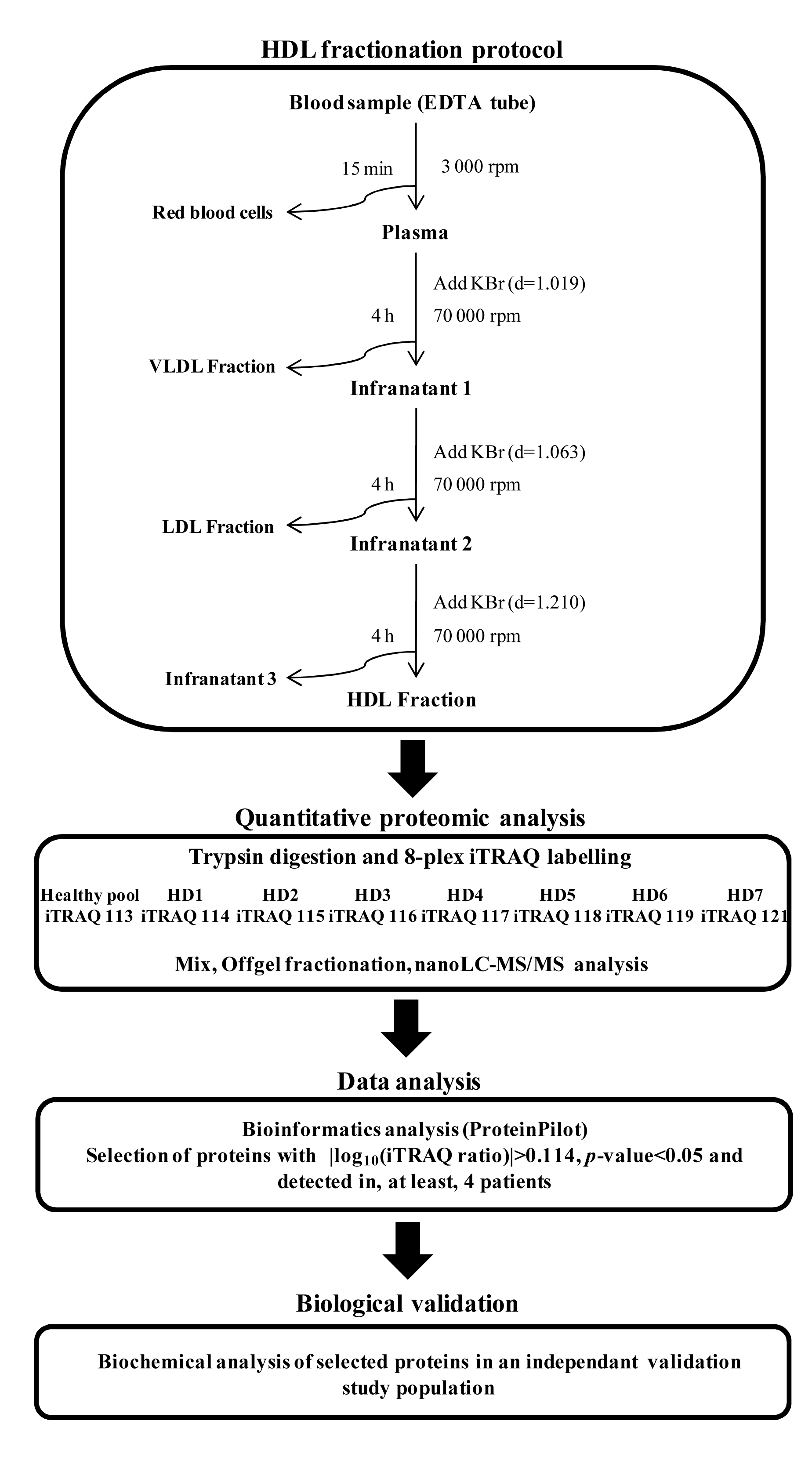

Supplement: Figure S1 — Experimental design. (TIF) [file pone.0034107.s001.tif]
