# Supplementary material for: HDL Proteome in Hemodialysis Patients: A Quantitative Nanoflow Liquid Chromatography-Tandem Mass Spectrometry Approach
Source: PLoS One. 2012 Mar 21;7(3):e34107. doi: 10.1371/journal.pone.0034107 (PMC3309955; doi:10.1371/journal.pone.0034107)
Supplement: Text S1 — (DOC) [file pone.0034107.s006.doc]

**Biochemical analyses – Performance of each assays**

The lower limit of detection (LLOD) for apoA-I and apoB were 0.25 g/l and 0.35 g/l respectively. Intra-assay coefficient of variation (CV) was 5.5% for apoA-I at 0.57 g/l and 3.2% at 0.56 g/l for apoB. Inter-assay CV was 5.8% for apoA-I at 0.57 g/l and 3.6% at 0.56 g/l for apoB. The LLOD were 0.01 g/L for serotransferin, 2 mg/L for apoC-II, 2 mg/L for apoC-III and 70 µg/L for haptoglobin. The intra-assay CV were 4.3% at 0.027 g/L for apoC-II, 1.4% at 0.037 g/L for apoC-III and 4.8% for haptoglobin. Inter-assay CV were 4.9% at 0.030 g/L for apoC-II, 1.44% at 0.039 g/L for apoC-III, 0.96% at 1.47 g/L for serotransferin and 7.8% for haptoglobin.

**iTRAQ Quantification**:

iTRAQ quantification was performed using the ProteinPilotTM software 2.0.1. All the quantitative ratios (both the average ratio for proteins and the individual peptide ratios) are corrected for bias. This correction accounts for pipetting error during the combination of the different labeled samples, based on the assumption that most proteins do not change in expression. The software identifies the median average protein ratio and corrects it to unity, and then applies this factor to all quantification results. The Pro Group™ Algorithm allows for a isoform specific quantification. ProteinPilot™ Software calculates protein ratios using only ratios from the spectra that are distinct to each protein (or protein form). This serves to eliminate any masking of changes in expression due to peptides that are shared between proteins. Only peptides with a check in the Used column in the Table S3 are used in the calculation of the average ratio for the protein.

The average iTRAQ ratio is inversely weighted by the % Error, a measure of the error in the calculated peptide ratio (the % Error is reported in the Table S3). The average ratio is calculated in log space so that apparent change will be the same regardless of how the ratio is defined. After the average ratio is calculated in log space, it is transformed back into linear space. The average ratio is calculated using the following equation:


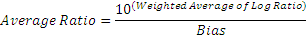


Weighted average of Log ratio is calculated using the following equation:


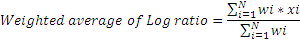


Where - x_i_= log (peptide ratio_i_), where (peptide ratio_i_) is the ratio for the ith observation.

- W_i_=1/% Error_i_, the weight for the ith observation.


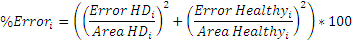


- N is the number of peptide ratios contributing to a protein’s average ratio.

The error factor for the average ratio is a statistic that has been created for reporting errors in ratios. It is a transformation of the 95% Confidence error from log space to linear space:


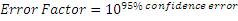


Where 95% Confidence Error = S_MW_ x (Student t Factor for N-1 degrees of freedom),

Where - N is the number of peptide ratios contributing to a protein’s average ratio.

- Student t factor come from table using the two-tailed 95% confidence column and degrees of freedom for N-1.

- S_MW_ is the weighted standard deviation of the weighted average of log ratios.

The weighted standard deviation is calculated using the following equation:


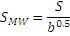


Where - S is the unweighted standard deviation


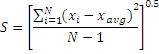


with x_i_=log(peptide ratio_i_) where (peptide ratio_i_) is the ratio for the ith observation; x_avg_ is the unweighted average of x_i_; and N is the number of peptide ratios contributing to a protein’s average ratio.

- b is the effective base


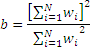


with w_i_=1/% Error_i_, the weighted for the ith observation, and N the number of peptide ratios contributing to a protein’s average ratio.

The significant cutoff threshold of fold change was determined using two equal amounts of tryptic digested protein samples labeled with iTRAQ reagent 114 and 115 respectively, and analyzed with 1D nanoLC/MS/MS. The standard deviation (S.D.) based on the ratios of the 80 identified proteins was 0.15. A cutoff of 1 ± 2 S.D. (1.3 and 0.77 for the significantly up-regulated and down-regulated proteins, respectively) was determined to be significant (p < 0.05).
